# Supplementary material for: A 3D gene expression atlas of the floral meristem based on spatial reconstruction of single nucleus RNA sequencing data
Source: Nat Commun. 2022 May 20;13:2838. doi: 10.1038/s41467-022-30177-y (PMC9122980; doi:10.1038/s41467-022-30177-y)
Supplement: Supplementary file 1 — Supplementary Information [file 41467_2022_30177_MOESM1_ESM.pdf]

Supplementary Figures

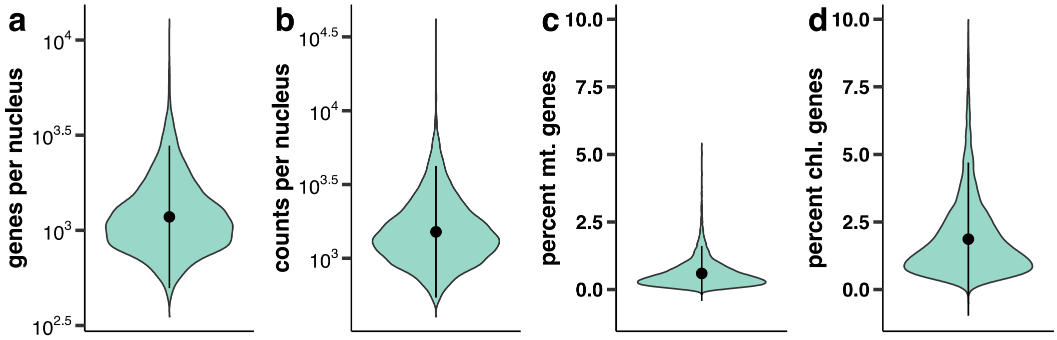

Supplementary Figure 1. **Summary of main statistics of the snRNA-seq.** **a** Number of expressed genes (containing at least one read) per nucleus. **b** Number of mapped reads per nucleus. **c** Percentage of reads mapping to the mitochondrial genome. **d** Percentage of reads mapping to the chloroplast genome. Error bars indicate standard deviation.

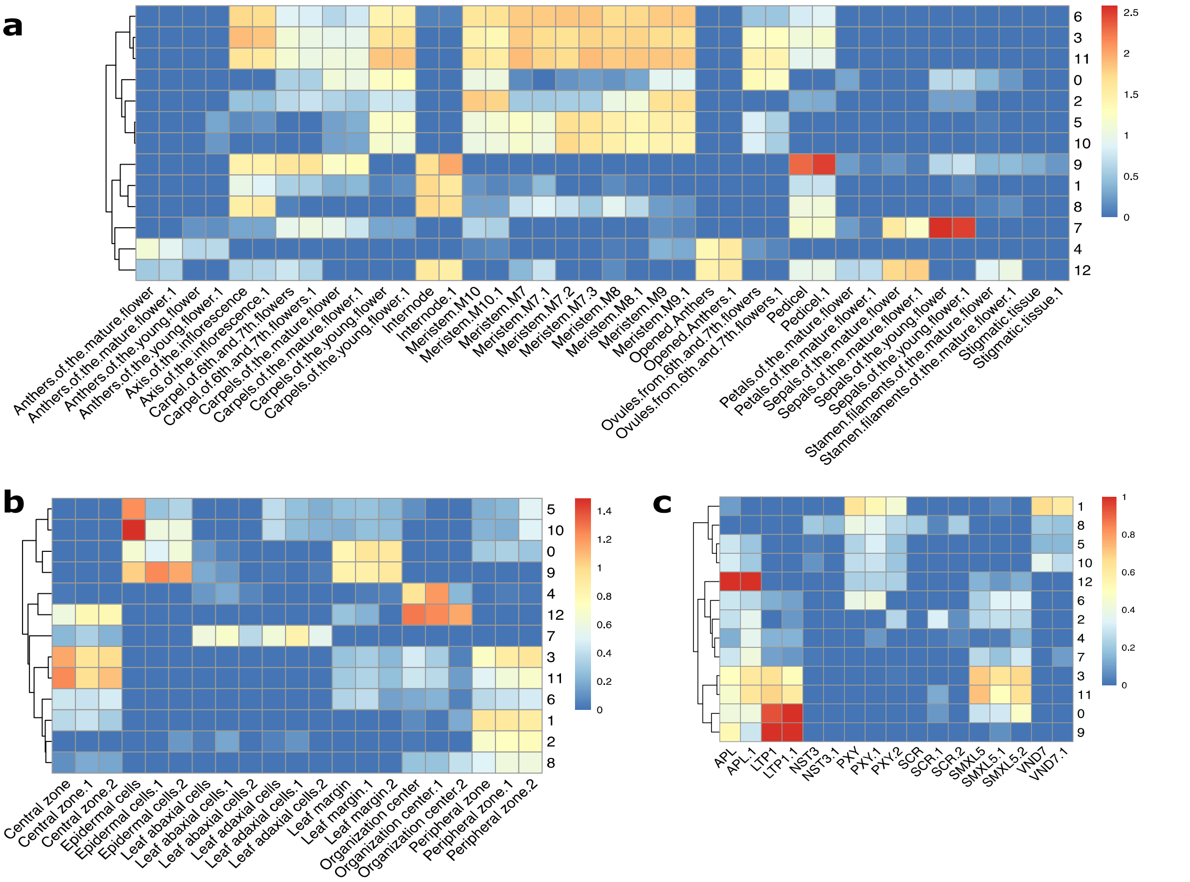

Supplementary Figure 2. **Average expression of the top 20 marker genes in publicly available bulk RNA-seq datasets.** Heatmaps show the expression of the top 20 significant marker genes for each snRNA-seq cluster in different publicly available bulk expression profiles of: several flower organs and developmental stages[1] (**a**), shoot apical meristem domains[2] (**b**), and vascular tissues of inflorescence stem[3] (**c**).

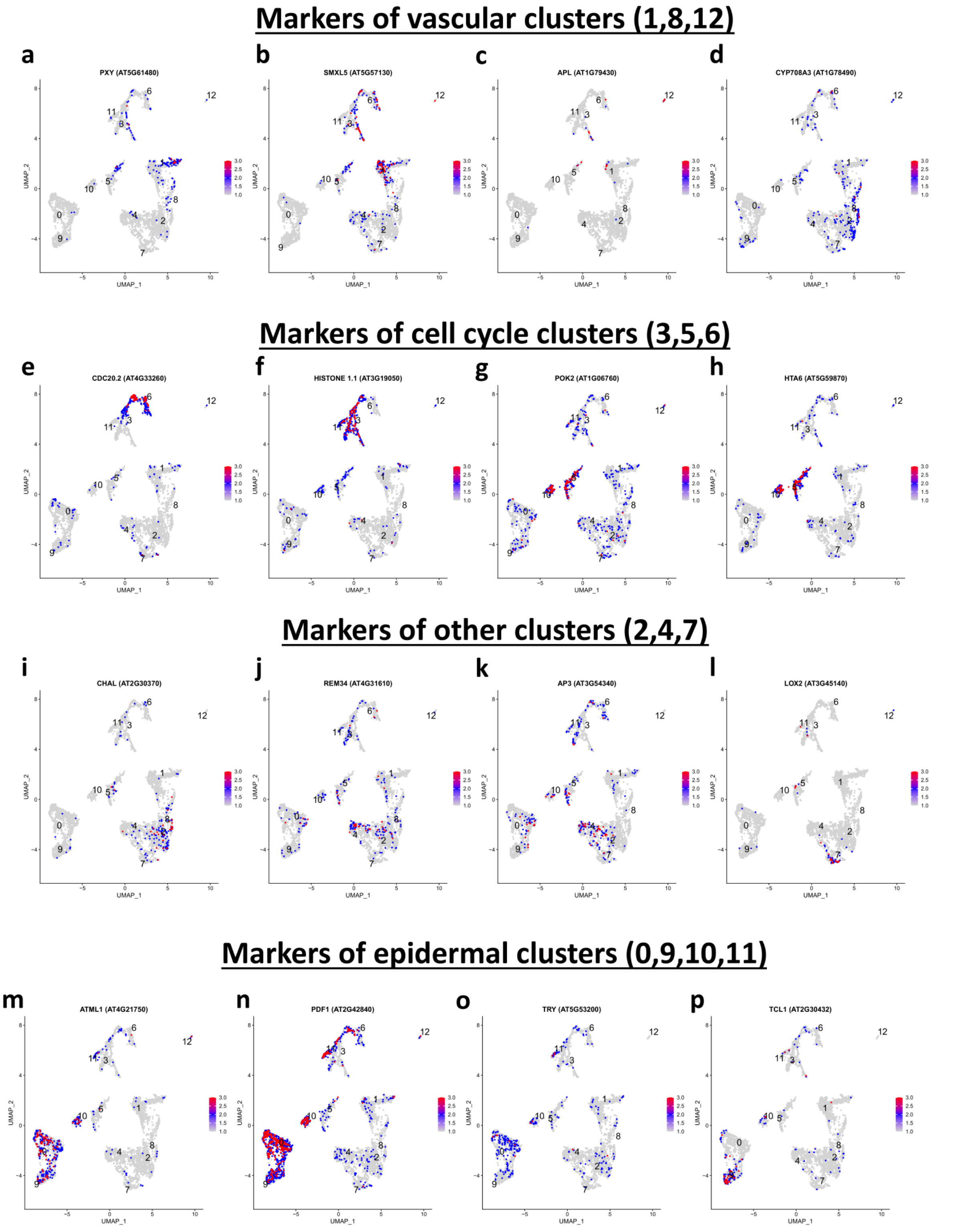

Supplementary Figure 3. **Expression of selected marker genes of snRNA-seq clusters on the UMAP plot.**

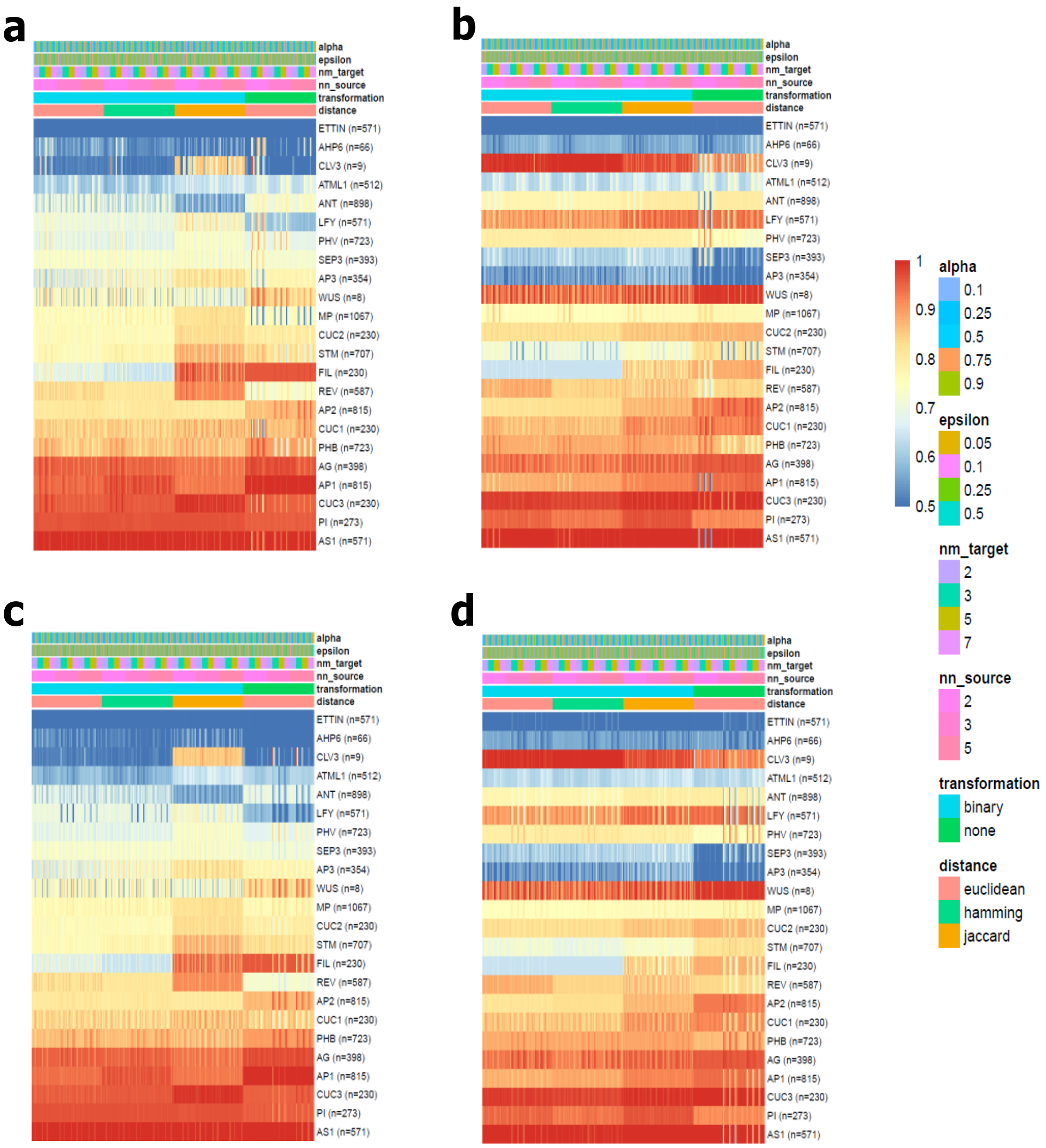

Supplementary Figure 4. **Gene-based performance of the method for gene expression reconstruction**. Heatmaps show the performance (AUROC) for each reference gene when that particular gene was removed from the spatial map during the data integration step. Four models were tested: **a** Filtering out snRNA-seq nuclei too dissimilar to the spatial map in the transcriptomic space (see Material and Methods) and using genes with high correlation with the reference genes in order to calculate transcriptomic distance among snRNA-seq nuclei (see Material and Methods). **b** Applying no filter to the snRNA-seq and using genes with high correlation with the reference genes in order to calculate transcriptomic distance among snRNA-seq nuclei. **c** Filtering out snRNA-seq nuclei too dissimilar to the spatial map in the transcriptomic space, and using the set of high variable genes defined by SEURAT to calculate

transcriptomic distances between snRNA-seq. **d** Applying no filter to the snRNA-seq data and using the set of high variable genes defined by SEURAT to calculate transcriptomic distances between snRNA-seq, this is the original option in novoSpaRc. The number between parentheses after the gene symbol indicates the number of cells where the particular gene is expressed in the spatial map. Legend indicates the different parameter values used for running novoSpaRc.

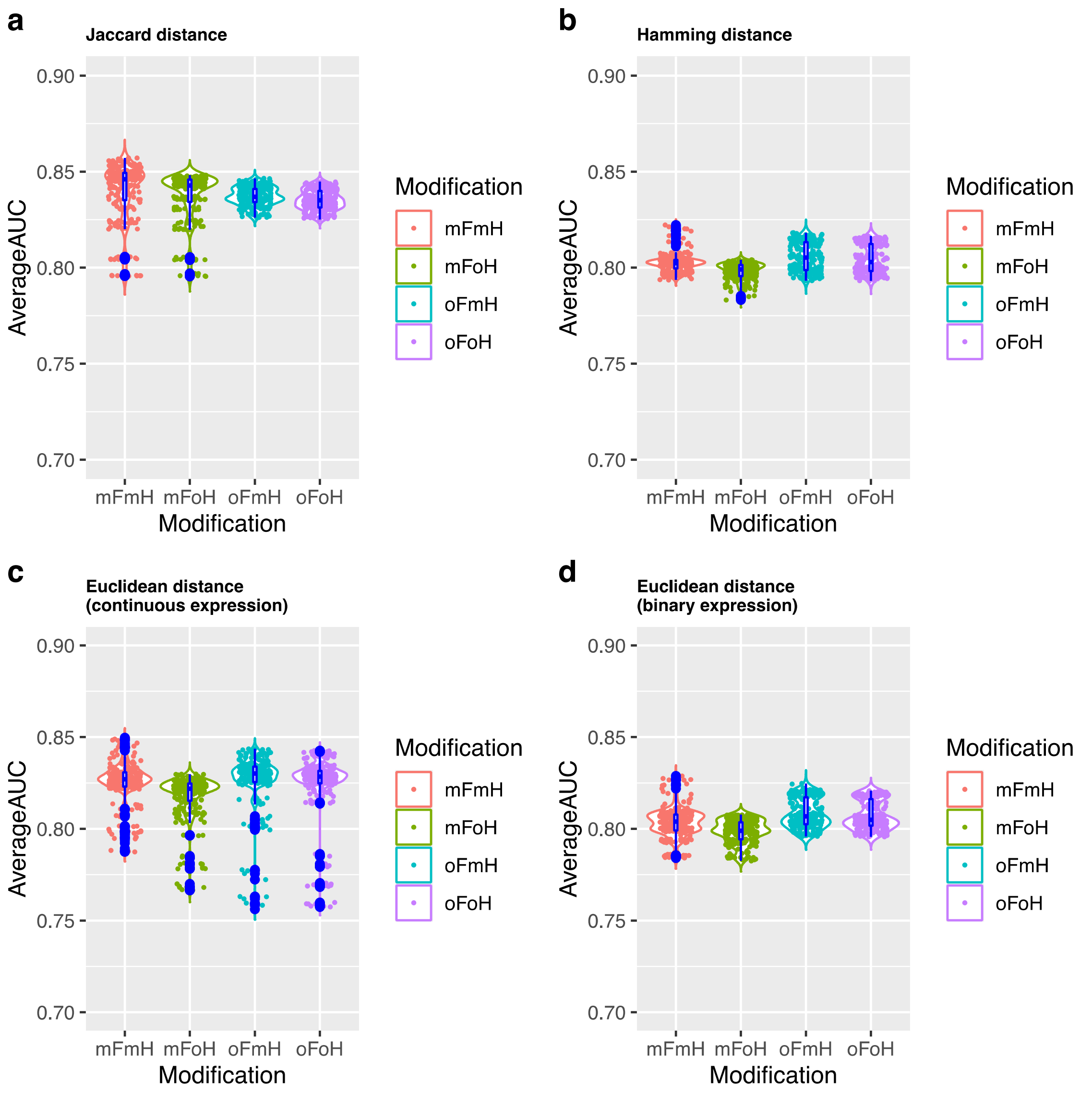

Supplementary Figure 5. **Average performance of our method for gene expression reconstruction**. Violin plots show the average AUROC values across all reference genes except ETTIN, AHP6, CLV3 and WUS, which were excluded because of their consistent low performance or because of the low number of cells where they are expressed. Four distances were tested: Jaccard (**a**), Hamming (**b**), Euclidean using snRNA-seq continuous expression (**c**) and Euclidean when the snRNA-seq data was binarized. For each distance, four models were tested: 1) Filtering out snRNA-seq nuclei too dissimilar to the spatial map in the transcriptomic space (see Material and Methods) and using genes with high correlation with the reference genes in order to calculate transcriptomic distance among snRNA-seq nuclei (mFmH). 2) Applying no filter to the snRNA-seq and using genes with high correlation with the reference

genes in order to calculate transcriptomic distance among snRNA-seq nuclei (oFmH). 3) Filtering out snRNA-seq nuclei too dissimilar to the spatial map in the transcriptomic space, and using the set of high variable genes defined by SEURAT to calculate transcriptomic distances between snRNA-seq (mFoH). 4) Applying no filter to the snRNA-seq data and using the set of high variable genes defined by SEURAT to calculate transcriptomic distances between snRNA-seq, this is the original option in novoSpaRc (oFoH). Box plots indicate median (middle line), 25th, 75th percentile (box) and 5th and 95th percentile (whiskers) as well as outliers (single points). In **a-d**, n=250 corresponding to the different parameter combinations used in Supplementary Figure 4.

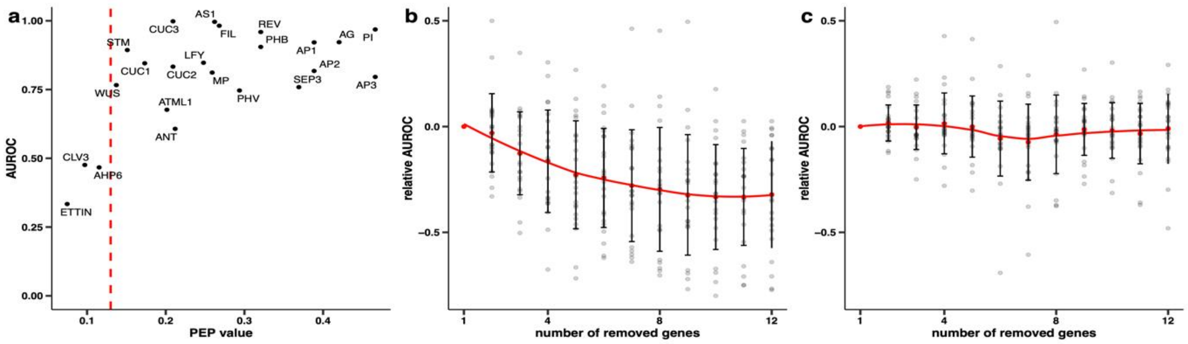

Supplementary Figure 6. **Performance of the reconstructed expression depends on PEP**. **a** Relationship between PEP and the performance (AUROC) of the gene expression estimation when the estimated gene was not included in the spatial map. Red line indicates the value 0.13. **b** Performance (AUROC) of the prediction for each gene (grey points) when x genes from the spatial map with highest co-expression values with the predicted gene were sequentially removed. **c** Performance (AUROC) of the prediction for each gene (grey points) when x genes from the spatial map with lowest co-expression values with the gene with the gene evaluated are removed. In **b** and **c**, the number of genes (x) removed is shown in the x-axis, and the drop in AUROC is shown in the y-axis; the red line represents a smoothing function (LOESS) applied to the average relative AUROC. Error bars indicate standard deviation (n=23 data points showed in grey)

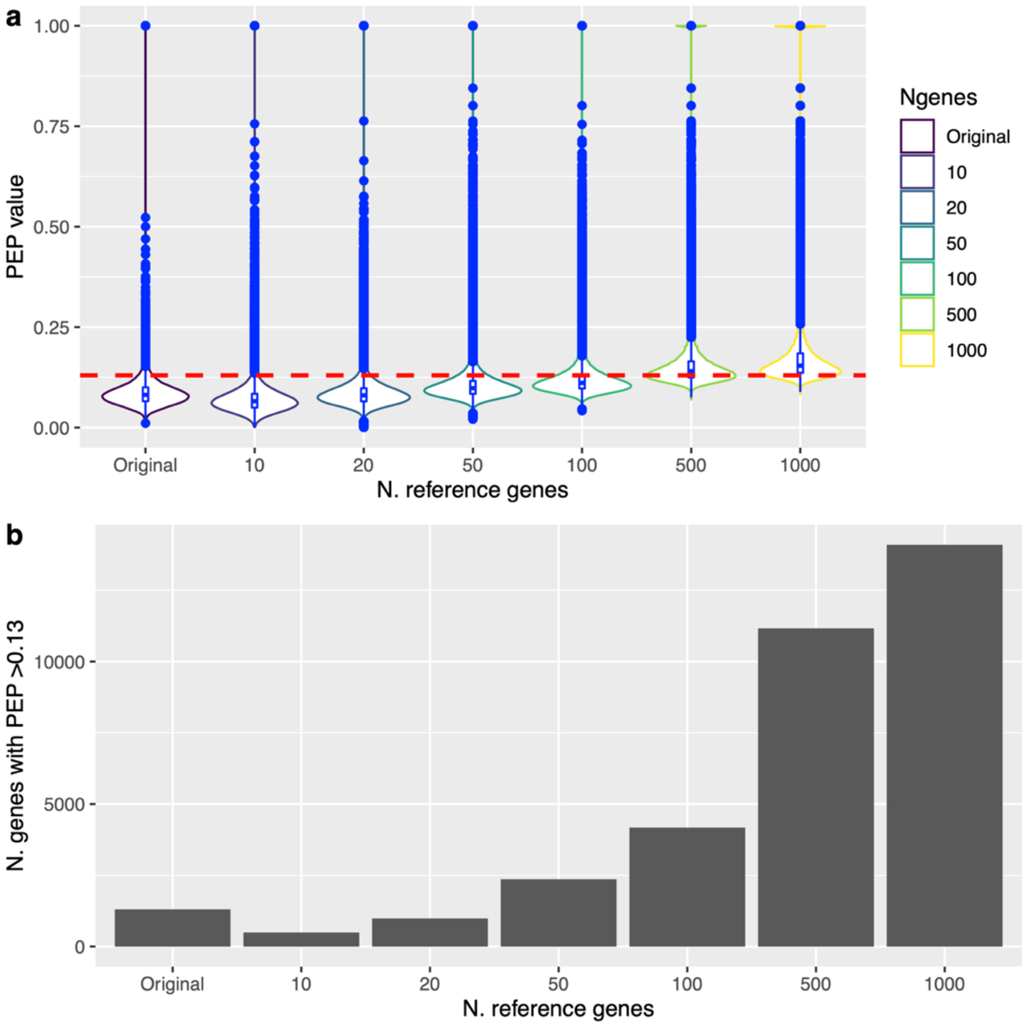

Supplementary Figure 7. **Increasing the number of reference genes increases the number of genes with PEP>0.13**. **a** PEP score distributions when using a random set on n reference genes (x-axis). **b** Average number of genes with a PEP-score>0.13 depending on the number of reference genes used (x-axis). Box plots indicate median (middle line), 25th, 75th percentile (box) and 5th and 95th percentile (whiskers) as well as outliers (single points).

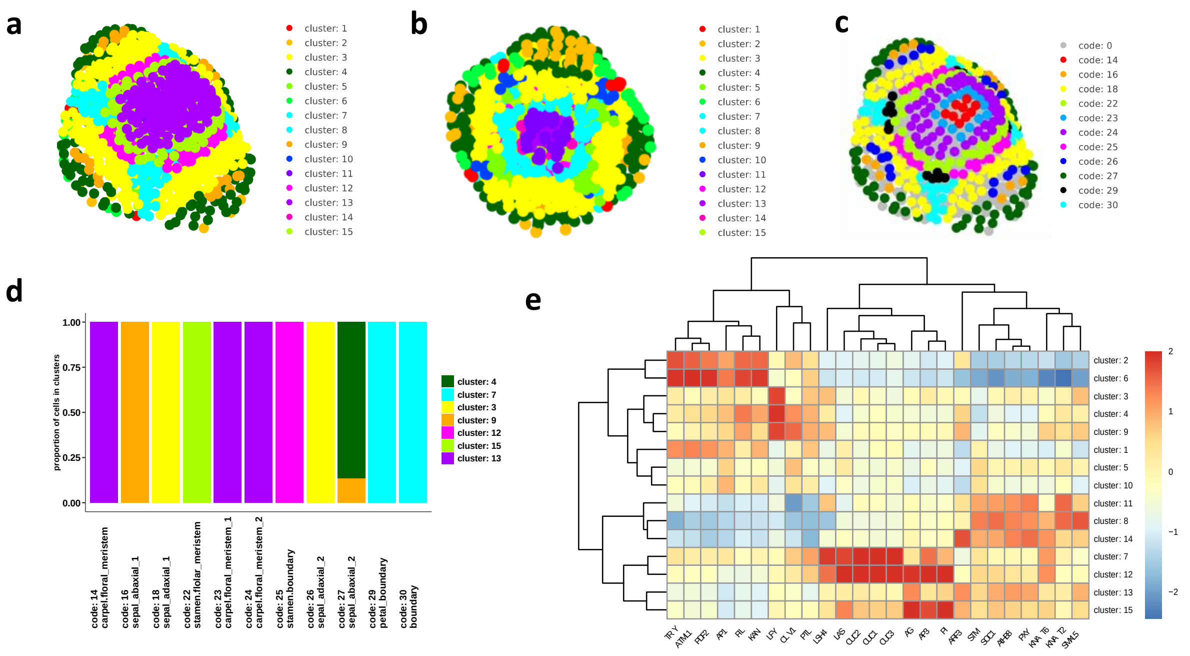

Supplementary Figure 8. **3D clustering of cells based on predicted gene expression recovers flower tissues.** Top (**a**) and bottom (**b**) view on the identified expression domains of the 3D meristem based on the predicted 3D gene expression profiles. **b** Expression domains as defined in Refahi *et al.*[4]. **c** Proportion of cells from expression domains of (**b**) in expression domains of (**a**). **e** Average relative expression of known floral markers in the identified expression domains from (**a**).

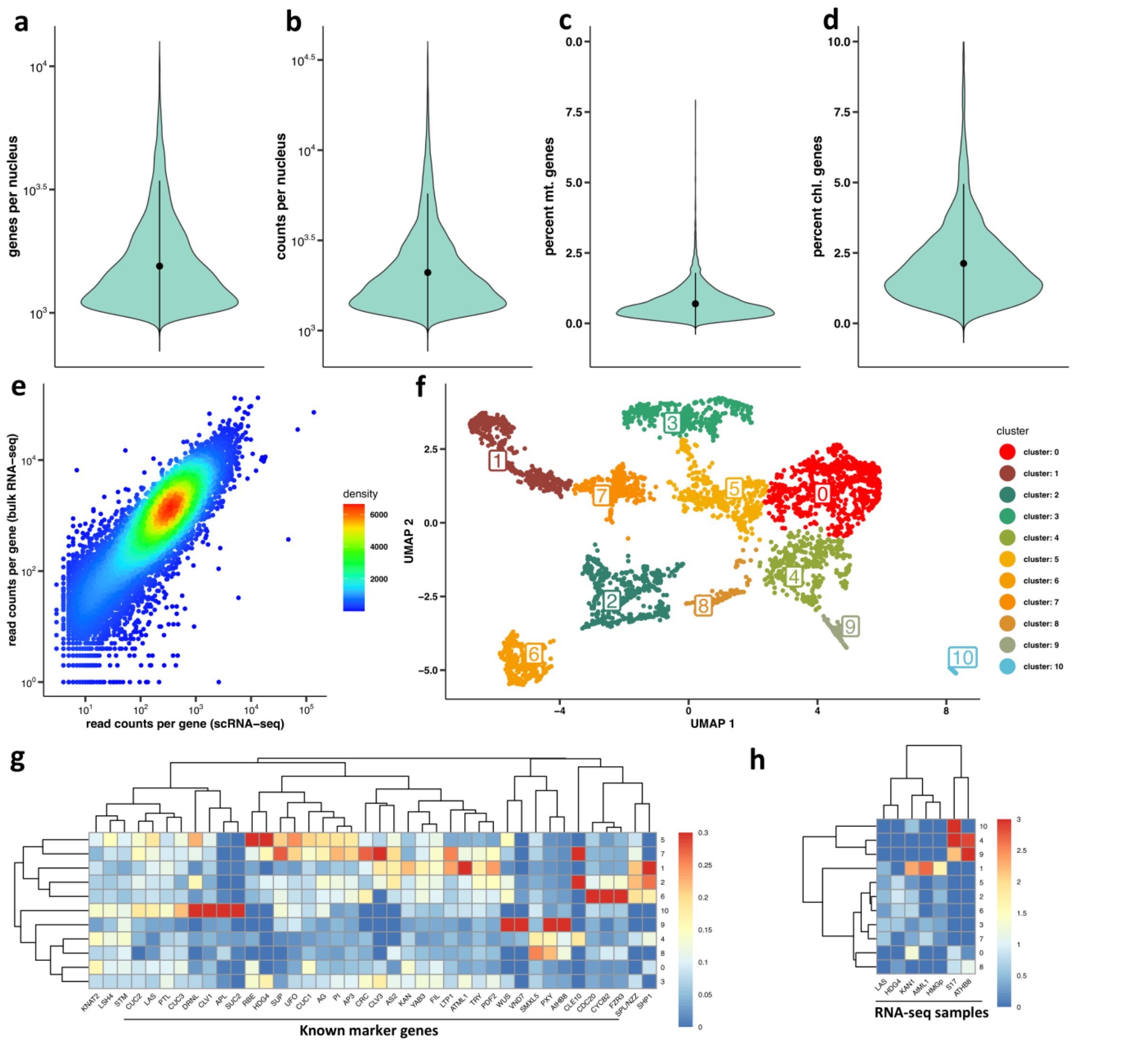

Supplementary Figure 9. **snRNA-seq analysis of the flower meristem after 3 days of DEX-induction.** **a** Number of expressed genes (containing at least one read) per nucleus. **b** Number of mapped reads per nucleus. **c** Percentage of reads mapping to the mitochondrial genome. **d** Percentage of reads mapping to the chloroplast genome. **e** Reproducibility (R= 0.88) of the gene expression estimated from computationally pooling all nuclei from our snRNA-seq (day 3) compared to bulk-RNA-seq of stage 5 flower meristem (day 4 after DEX-induction, average of 3 biological replicates). **f** UMAP plot and clustering snRNA-seq analysis of Arabidopsis floral meristems obtained by Seurat analysis. **g** Average relative expression of known floral markers on the identified snRNA-seq clusters. **h** Relationship between domain-specific shoot apical meristem bulk RNA-seq datasets profiled by Tian *et al.*[5] and the snRNA-seq clusters. The heatmap shows the relative average expression of the top 20 marker genes for each snRNA-seq cluster (y-axis) on domain-specific shoot apical meristem bulk RNA-seq datasets. In **a-d**, error bars indicate standard deviation.

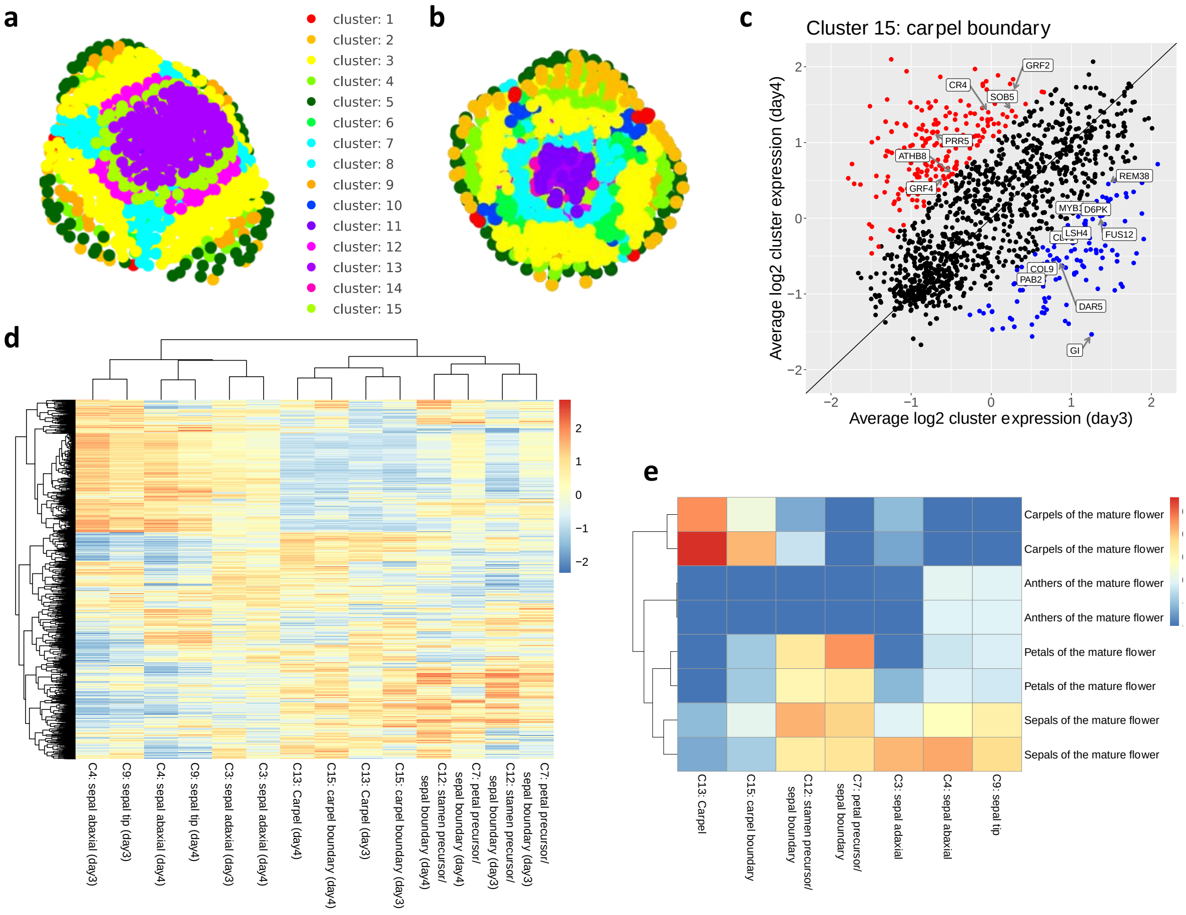

Supplementary Figure 10. **Gene expression changes between time-points.** Top (**a**) and bottom (**b**) view on the identified expression-domains of the 3D meristem based on the predicted gene expression profiles of day 3 after DEX-induction snRNA-seq sample. **d** Heatmap showing the average gene expression for each expression-domain at the different time-points considered. **c** Scatterplot of the average gene expression in the domain "15: carpel boundary" at the two time-points considered. Red indicates genes that have a change in expression bigger than 1 log2 fold-change when comparing day 4 versus day 3, and blue are the genes with a log2 fold-change expression lower than -1. Some important flower regulators are shown in the graph as examples. **e** Relative median expression of the genes upregulated (log2 fold-change>1) in day 4 versus day 3 for each identified domain (columns) in the mature flower organ (rows; data obtained from Klepikova *et al.*[6])

a b

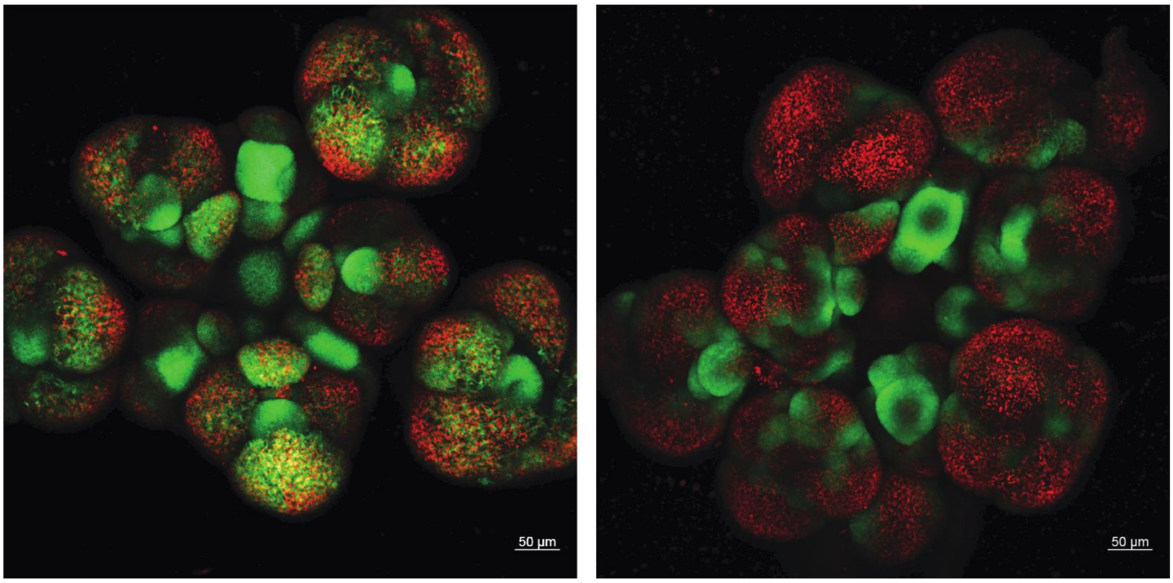

Supplementary Figure 11. **GFP signals in pAGi::NTF (a) and pAP3::NTF(a) domain specific lines used for FANS.** Scale bars indicate 50 µm. The visualized expression patterns match with published expression data of AG (Ito *et al.*[7]) and AP3 (Prunet *et al.*[8]). The experiment was performed at least twice for one representative line each.

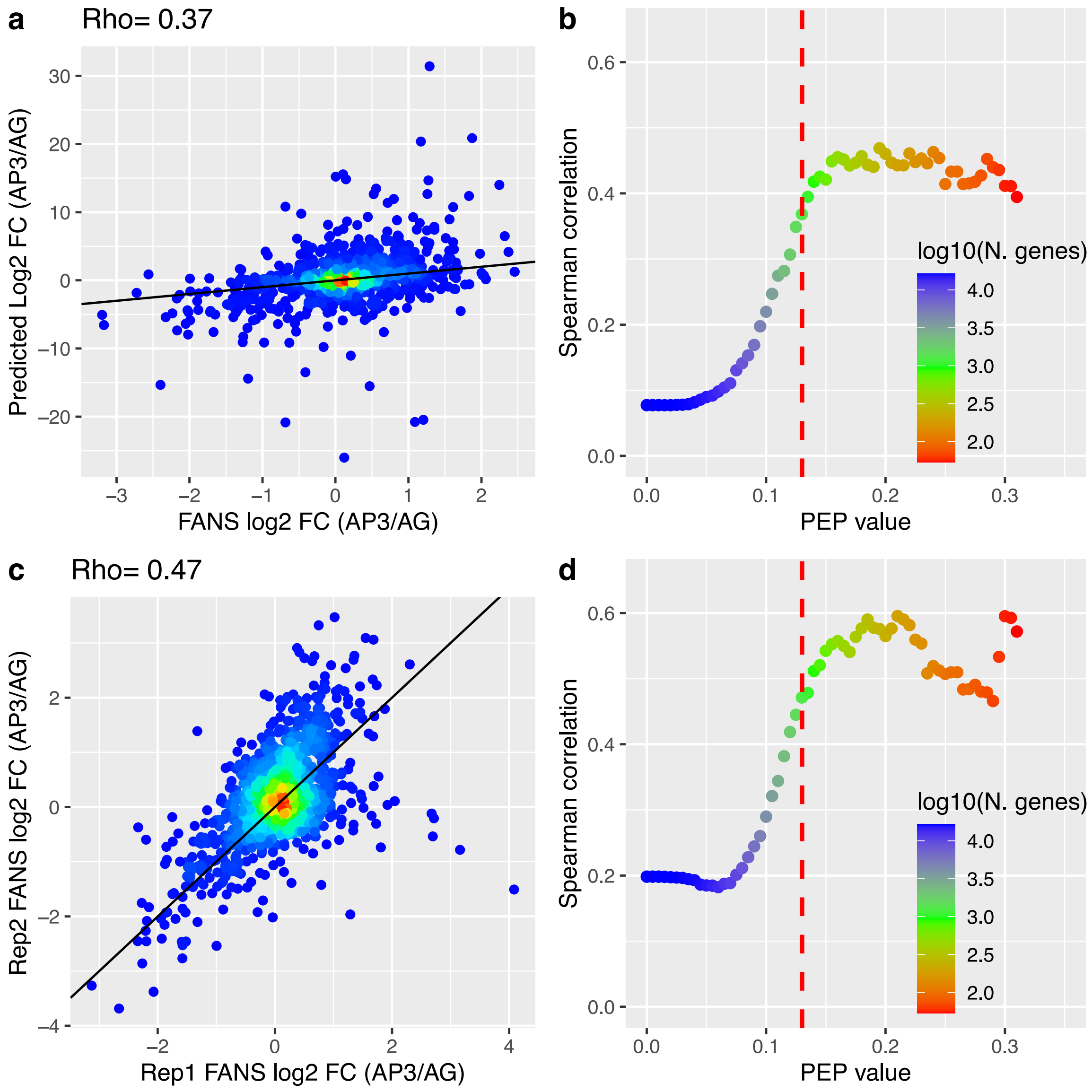

Supplementary Figure 12. **Prediction of AP3 vs AG domain-specific log2FC expression. a** Scatterplot showing the predicted change in expression between the AP3 and AG domain predicted by our method (y-axis) and observed by our bulk FANS RNA-seq data (x-axis) when using genes with PEP>0.13 (n=1,306). Continuous black line indicates the diagonal line. The associated Spearman correlation is 0.37. The associated spearman correlation for other values of PEP can be seen in **b**. Bottom row shows the scatterplot for the observed change in expression of both biological FANS bulk RNA-seq replicates for AP3 versus AG. Color in **b** and **c** indicates the number of genes predicted at this level of PEP. Vertical red line indicates the value of 0.13.

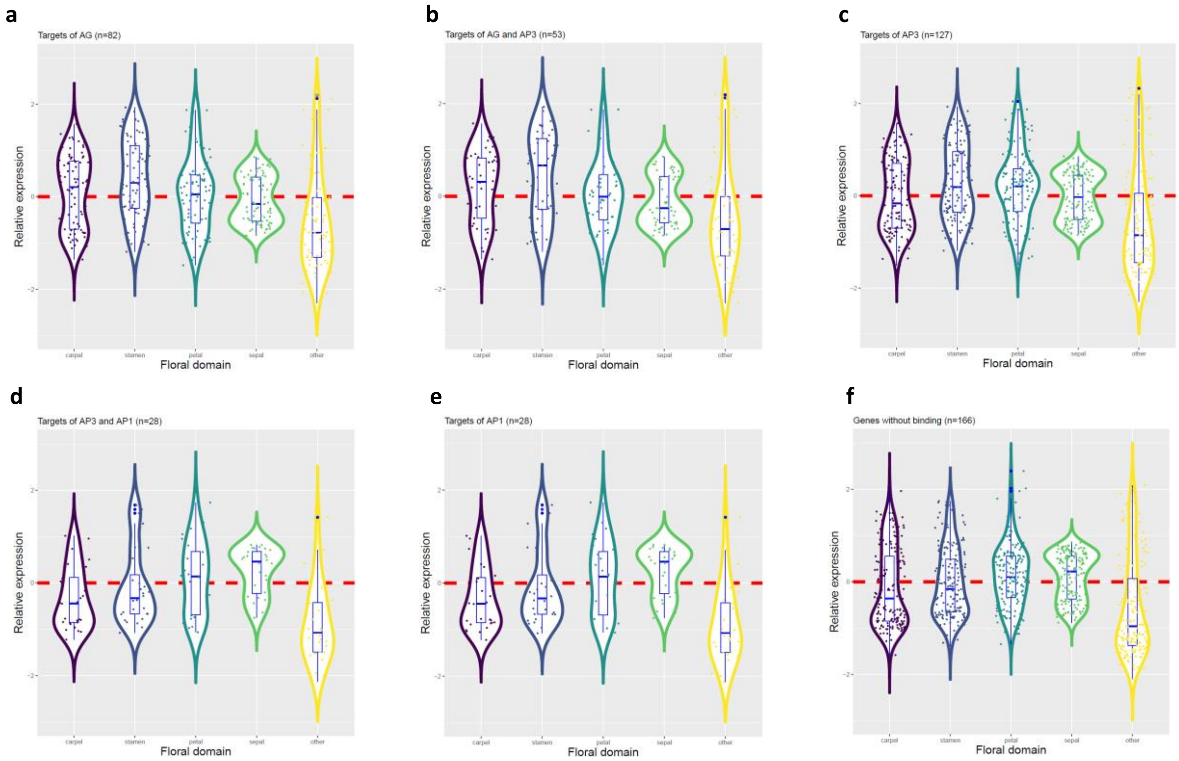

Supplementary Figure 13. **Gene expression distribution in the floral meristem whorls depending on TF binding.** Gene expression was standardized to mean 0 variance 1, after average expression was calculated for each gene in the different floral whorls. Floral whorls are defined as: carpel: cells expressing AG but not AP3 neither AP1; stamen: cells expressing AG and AP3 but not AP1, petals: cells expressing AP3 and AP1 but not AG; sepals: cells expressing AP1 but not AG neither AP3. Four groups of genes were considered: **a** genes with a AG binding in the gene body or the 2 kb regions around, **b** genes with AG and AP3 binding, **c** genes with an AP3 binding, **d** genes with an AP3 and AP1 binding, **e** genes with an AP1 binding and **f** genes without any AG, AP3, or AP1 binding. Note that binding events of several TFs to the same gene does not necessitate that these TFs bind as part of the same complex, their binding could be independent and occur in different cells. Box plots indicate median (middle line), 25th, 75th percentile (box) and 5th and 95th percentile (whiskers) as well as outliers (single points). In the top of each figure, it is indicated the number of points (target genes) used.

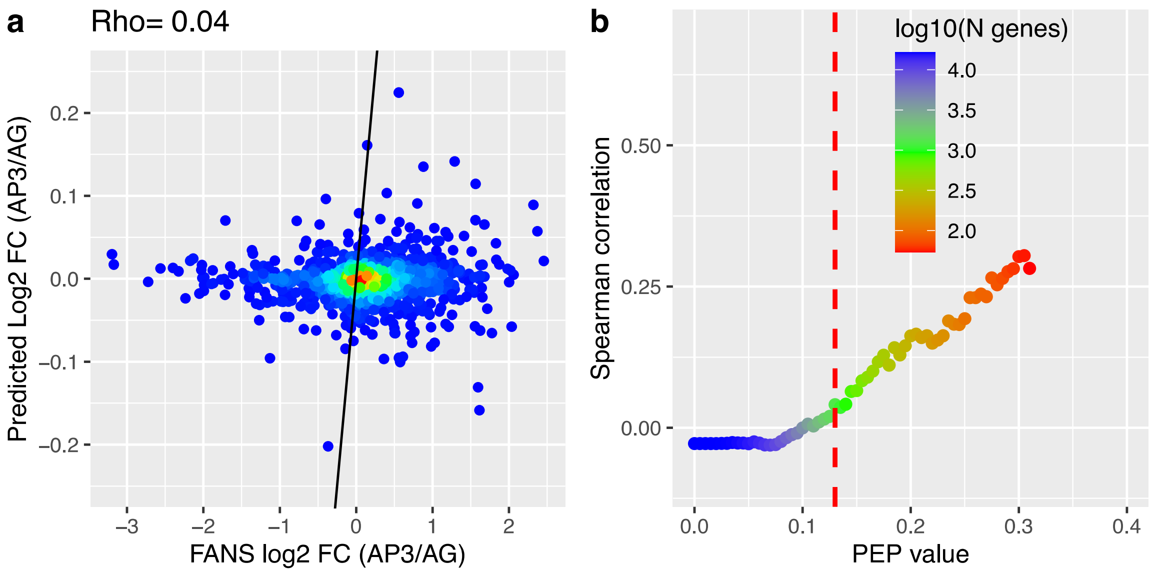

Supplementary Figure 14. **Prediction of AP3 vs AG domain-specific log2 FC expression directly from snRNA-seq. a** Scatterplot showing the predicted change in expression between the AP3 and AG domains predicted directly by the snRNA-seq (y-axis) or observed by our bulk RNA-seq data (x-axis) when using genes with PEP>0.13 (n=1,306). The associated Spearman correlation is 0.04 (pv< 0.14; not significant). The associated spearman correlation for other values of PEP can be seen in (**b**). Vertical red line indicates the value of 0.13.

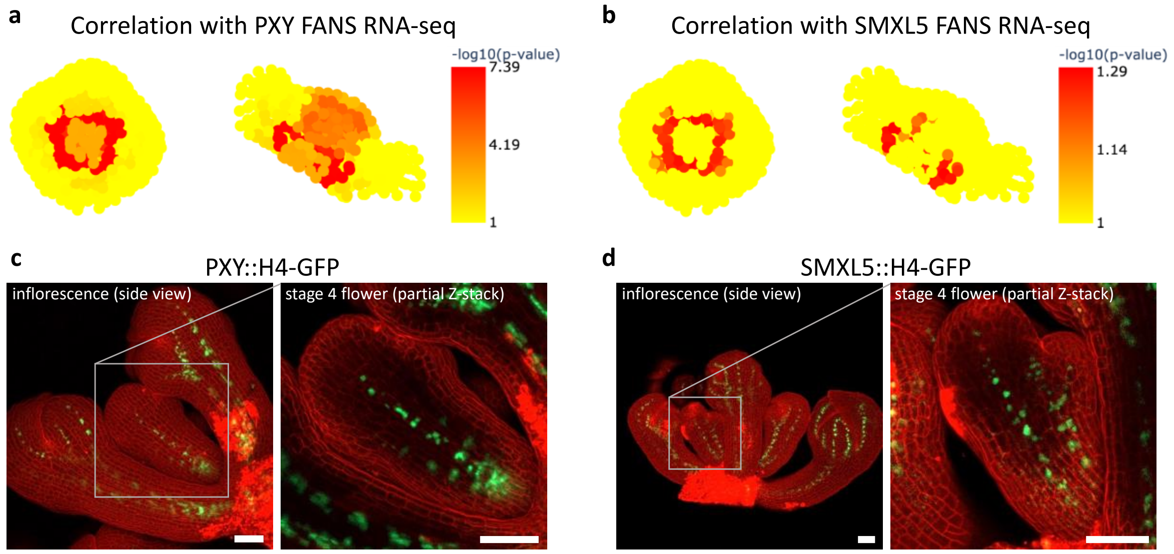

Supplementary Figure 15. **Localization of the vascular stem cells in the flower meristem**. Predicted location of the vascular stem cells was calculated by the -log10 p-value of the Pearson correlation for different vascular FANS RNA-seq datasets (PXY: **a**, and SMXL5: **b**) to the reconstructed transcriptomes of each cell of the spatial map. **c** and **d** show H4-GFP expression (green) driven by the PXY and by the SMXL5 promoter, respectively. Images display side views of an inflorescence (left) and a stage 4 flower (right). For improved visualization of the GFP signal within the pedicel, the top layers of the Z-stack were removed from the orthogonal projection. Cell walls were stained using propidium iodide (red). Scale bars indicate 50 µm. PXY and SMXL5 promoter-reporter lines have been described previously by Shi et al.[9] The experiment was performed at least twice for one representative line each.

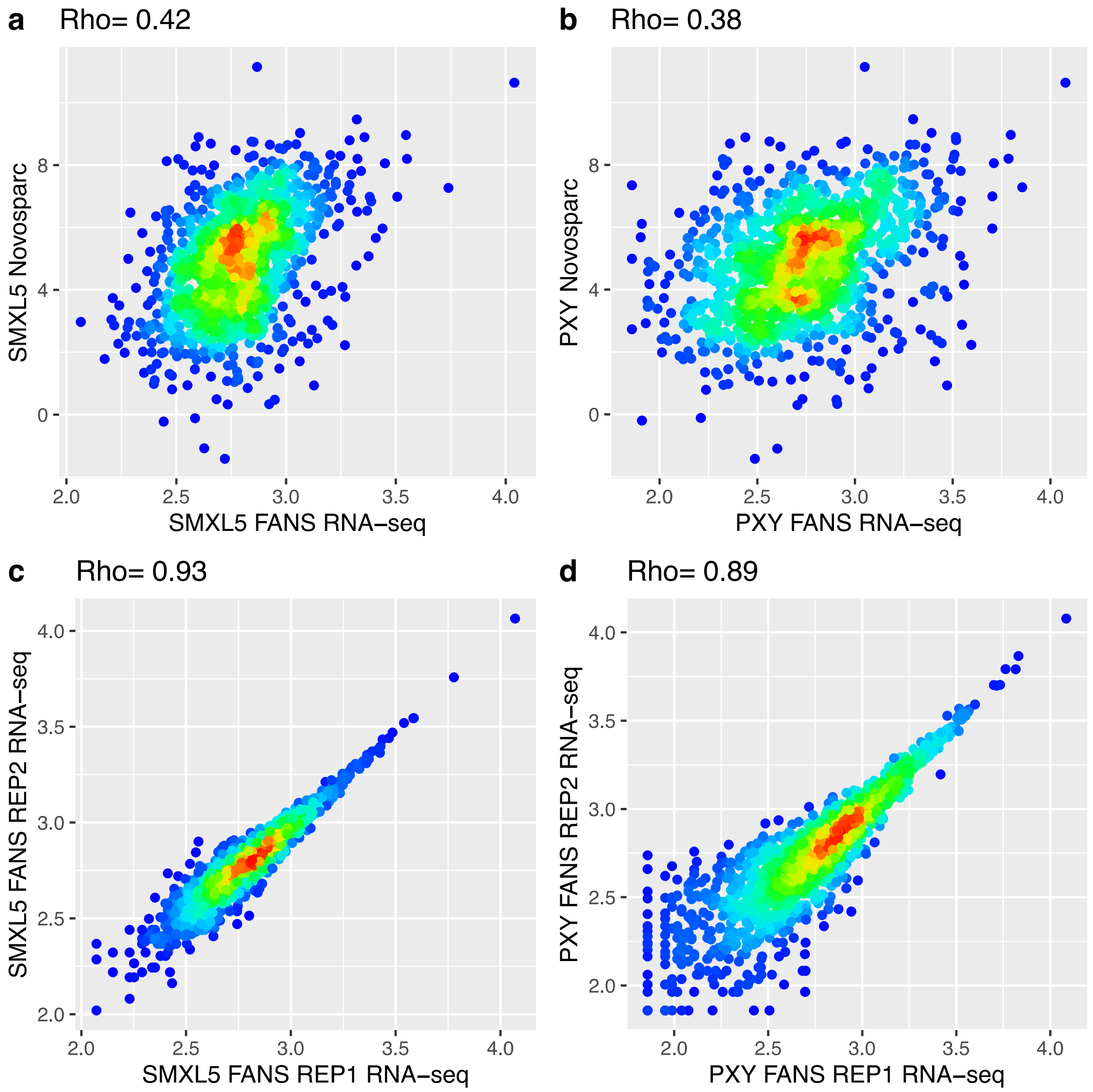

Supplementary Figure 16. **Prediction of vascular domain-specific expression.** Scatterplot showing the gene expression for SMXL5 (**a**), and PXY (**b**) domain predicted by our method (y-axis) and observed by publicly available FANS bulk RNA-seq data (x-axis) when using genes with PEP value>0.13 (n=1,306). Bottom row shows the scatterplot for the gene expression of both biological FANS bulk RNA-seq replicates for SMXL5 (**c**), and PXY (**d**).

Supplementary References

1. Klepikova, A. V, Kasianov, A. S., Gerasimov, E. S., Logacheva, M. D. & Penin, A. A. A high resolution map of the Arabidopsis thaliana developmental transcriptome based on RNA-seq profiling. *The Plant Journal* **88**, 1058–1070 (2016).
2. Tian, C. *et al.* A gene expression map of shoot domains reveals regulatory mechanisms. *Nature Communications* **10**, 141 (2019).
3. Shi, D. *et al.* Tissue-specific transcriptome profiling of the Arabidopsis inflorescence stem reveals local cellular signatures. *The Plant Cell* **33**, 200–223 (2021).
4. Refahi, Y. *et al.* A multiscale analysis of early flower development in *Arabidopsis* provides an integrated view of molecular regulation and growth control. *Developmental Cell* **56**, 540-556.e8 (2021).
5. Tian, C. *et al.* A gene expression map of shoot domains reveals regulatory mechanisms. *Nature Communications* **10**, 141 (2019).
6. Klepikova, A. v, Kasianov, A. S., Gerasimov, E. S., Logacheva, M. D. & Penin, A. A. A high resolution map of the Arabidopsis thaliana developmental transcriptome based on RNA-seq profiling. *The Plant Journal* **88**, 1058–1070 (2016).
7. Ito, T. *et al.* The homeotic protein AGAMOUS controls microsporogenesis by regulation of *SPOROCYTELESS*. *Nature* **430**, 356–360 (2004).
8. Nathanaël, P., Weibing, Y., Pradeep, D., M, M. E. & P, J. T. *SUPERMAN* prevents class B gene expression and promotes stem cell termination in the fourth whorl of *Arabidopsis* thaliana flowers. *Proceedings of the National Academy of Sciences* **114**, 7166–7171 (2017).
9. Shi, D. *et al.* Tissue-specific transcriptome profiling of the Arabidopsis inflorescence stem reveals local cellular signatures. *The Plant Cell* **33**, 200–223 (2021).
