# Supplementary material for: A 3D gene expression atlas of the floral meristem based on spatial reconstruction of single nucleus RNA sequencing data
Source: Nat Commun. 2022 May 20;13:2838. doi: 10.1038/s41467-022-30177-y (PMC9122980; doi:10.1038/s41467-022-30177-y)
Supplement: Supplementary file 3 — Description of Additional Supplementary Files [file 41467_2022_30177_MOESM3_ESM.pdf]

Description of Additional Supplementary Files

File Name: Supplementary Data 1
Description: Gene expression prediction in the floral whorls

File Name: Supplementary Data 2
Description: UMAP cluster annotations and markers

File Name: Supplementary Data 3
Description: Program for gentleMACS Dissociator
