# Supplementary material for: A 3D gene expression atlas of the floral meristem based on spatial reconstruction of single nucleus RNA sequencing data
Source: Nat Commun. 2022 May 20;13:2838. doi: 10.1038/s41467-022-30177-y (PMC9122980; doi:10.1038/s41467-022-30177-y)
Supplement: Supplementary file 7 — Reporting Summary [file 41467_2022_30177_MOESM7_ESM.pdf]

nature portfolio

Corresponding author(s): Jose M Muino

Last updated by author(s): Apr 12, 2022

# Reporting Summary

Nature Portfolio wishes to improve the reproducibility of the work that we publish. This form provides structure for consistency and transparency in reporting. For further information on Nature Portfolio policies, see our Editorial Policies and the Editorial Policy Checklist.

## Statistics

For all statistical analyses, confirm that the following items are present in the figure legend, table legend, main text, or Methods section.

| n/a | Confirmed | |
|---|---|---|
| ☒ | ☐ | The exact sample size (*n*) for each experimental group/condition, given as a discrete number and unit of measurement |
| ☐ | ☒ | A statement on whether measurements were taken from distinct samples or whether the same sample was measured repeatedly |
| ☐ | ☒ | The statistical test(s) used AND whether they are one- or two-sided<br>*Only common tests should be described solely by name; describe more complex techniques in the Methods section.* |
| ☒ | ☐ | A description of all covariates tested |
| ☐ | ☒ | A description of any assumptions or corrections, such as tests of normality and adjustment for multiple comparisons |
| ☐ | ☒ | A full description of the statistical parameters including central tendency (e.g. means) or other basic estimates (e.g. regression coefficient) AND variation (e.g. standard deviation) or associated estimates of uncertainty (e.g. confidence intervals) |
| ☐ | ☒ | For null hypothesis testing, the test statistic (e.g. *F*, *t*, *r*) with confidence intervals, effect sizes, degrees of freedom and *P* value noted<br>*Give P values as exact values whenever suitable.* |
| ☒ | ☐ | For Bayesian analysis, information on the choice of priors and Markov chain Monte Carlo settings |
| ☒ | ☐ | For hierarchical and complex designs, identification of the appropriate level for tests and full reporting of outcomes |
| ☐ | ☒ | Estimates of effect sizes (e.g. Cohen's *d*, Pearson's *r*), indicating how they were calculated |

*Our web collection on statistics for biologists contains articles on many of the points above.*

## Software and code

Policy information about availability of computer code

| | |
|---|---|
| Data collection | CellRanger v3.1.0 |
| Data analysis | R v3.6<br>Seurat v3.2.3<br>Trimmomatic v0.36<br>STAR v2.7.0b<br>DESeq2 v1.24.0<br>featureCounts v1.6.4<br>ZEISS Zen blue 2.1 |

For manuscripts utilizing custom algorithms or software that are central to the research but not yet described in published literature, software must be made available to editors and reviewers. We strongly encourage code deposition in a community repository (e.g. GitHub). See the Nature Portfolio guidelines for submitting code & software for further information.

## Data

Policy information about availability of data

All manuscripts must include a data availability statement. This statement should provide the following information, where applicable:

- Accession codes, unique identifiers, or web links for publicly available datasets
- A description of any restrictions on data availability
- For clinical datasets or third party data, please ensure that the statement adheres to our policy

The snRNA-seq and bulk RNA-seq data have been deposited in the GEO database under accession number GSE174599 and GSE174656, respectively.

# Field-specific reporting

Please select the one below that is the best fit for your research. If you are not sure, read the appropriate sections before making your selection.

☒ Life sciences ☐ Behavioural & social sciences ☐ Ecological, evolutionary & environmental sciences

For a reference copy of the document with all sections, see nature.com/documents/nr-reporting-summary-flat.pdf

# Life sciences study design

All studies must disclose on these points even when the disclosure is negative.

| | |
|---|---|
| Sample size | For bulk RNA-seq, we used 3 biological replicates as typical in the field |
| Data exclusions | no data was excluded |
| Replication | Main statment of the snRNA-seq analysis was validated with microscopy analysis of specific reporter genes |
| Randomization | The samples were collected randomly |
| Blinding | N/a |

# Reporting for specific materials, systems and methods

We require information from authors about some types of materials, experimental systems and methods used in many studies. Here, indicate whether each material, system or method listed is relevant to your study. If you are not sure if a list item applies to your research, read the appropriate section before selecting a response.

## Materials & experimental systems

| n/a | Involved in the study | |
|---|---|---|
| ☒ | ☐ | Antibodies |
| ☒ | ☐ | Eukaryotic cell lines |
| ☒ | ☐ | Palaeontology and archaeology |
| ☒ | ☐ | Animals and other organisms |
| ☒ | ☐ | Human research participants |
| ☒ | ☐ | Clinical data |
| ☒ | ☐ | Dual use research of concern |

## Methods

| n/a | Involved in the study | |
|---|---|---|
| ☒ | ☐ | ChIP-seq |
| ☐ | ☒ | Flow cytometry |
| ☒ | ☐ | MRI-based neuroimaging |

## Flow Cytometry

### Plots

Confirm that:

☒ The axis labels state the marker and fluorochrome used (e.g. CD4-FITC).

☒ The axis scales are clearly visible. Include numbers along axes only for bottom left plot of group (a 'group' is an analysis of identical markers).

☒ All plots are contour plots with outliers or pseudocolor plots.

☒ A numerical value for number of cells or percentage (with statistics) is provided.

### Methodology

| | |
|---|---|
| Sample preparation | Inflorescences were gently crushed to pieces in liquid nitrogen using a mortar and a pestle and then transferred to a gentleMACS M tube. After liquid nitrogen evaporated totally, 5 ml of Honda buffer (2.5% Ficoll 400, 5% Dextran T40, 0.4 M sucrose, 10 mM $MgCl_2$, 1 µM DTT, 0.5% Triton X-100, 1 tablet/50 ml cOmplete Protease Inhibitor Cocktail, 0.4 U/µl RiboLock, 25 mM Tris-HCl, pH 7.4) was added to the tube. Nuclei were released at 4 °C by homogenizing the tissue on a gentleMACS Dissociator with a running program as described previously (Sunaga-Franze et al. 2021). The resulting homogenate was filtered through a 70 µm strainer, and another 5ml Honda buffer was applied onto the filter to collect the remaining nuclei. Nuclei were then pelleted by centrifugation at 1000 g for 6 min at 4 °C and then resuspended gently in 500 µl Honda buffer. The nuclei suspension was filtered again through a 30 µm strainer, diluted by adding 500 µl PBS buffer, and stained with 2 µM DAPI. Ambion RNase Inhibitor and SUPERaseIn RNase Inhibitor were added to a final concentration of 0.4 U/µl and 0.2 U/µl, respectively. 200,000 events of single nuclei were selected on DAPI signals by a BD FACS Aria Fusion into a 1.5-ml tube with landing buffer (15 µl 4% BSA in PBS with 80 U Ambion RNase Inhibitor and 80 U SUPERaseIn RNase Inhibitor). |

| | |
|---|---|
| Instrument | BD FACSAria Fusion |
| Software | FACSDiVa v8.0.2 |
| Cell population abundance | The nuclei population is around 15% of total events for the 4 Day inflorescences and 50% for the 3 Day inflorescences. The purity of nuclei is determined by the highest intensity and sharp peaks of DAPI signal. Sample without DAPI was used as a control to set the DAPI gate to exclude auto-florescent components. |
| Gating strategy | Nuclei population cannot be distinguished by the preliminary SSC and FSC gate only, so outliers from the main population with extreme values (supposedly small debris and larger aggregates) are excluded. Nuclei doublets off the diagonal of the FSC-H vs FSC-A and SSC-H vs SSC-A plots are excluded. Nuclei with the highest DAPI intensity (two peaks representing 2c or 4 c of DNA amount ) in the DAPI-H vs DAPI-A plot are selected, while damaged nuclei containing only DNA/RNA residues with low DAPI signal are excluded. |

☒ Tick this box to confirm that a figure exemplifying the gating strategy is provided in the Supplementary Information.
